# Combining greedy and evolutionary algorithms to maximize influence in networks under deterministic linear threshold model

**DOI:** 10.1371/journal.pone.0331109

**Published:** 2025-09-08

**Authors:** Alexander Andreev, Stepan Kochemazov, Alexander Semenov

**Affiliations:** 1 Information Technologies and Programming Faculty, ITMO University, Saint Petersburg, Russia; 2 Laboratory of Logical and Optimization Methods for Analysis of Complex Systems, Matrosov Institute for System Dynamics and Control Theory SB RAS, Irkutsk, Russia; Shanghai Jiao Tong University - Xuhui Campus, CHINA

## Abstract

In the paper we consider the well-known Influence Maximization (IM) and Target Set Selection (TSS) problems for Boolean networks under Deterministic Linear Threshold Model (DLTM). The main novelty of our paper is that we state these problems in the context of pseudo-Boolean optimization and solve them using evolutionary algorithms in combination with the known greedy heuristic. We also propose a new variant of (1 + 1)-Evolutionary Algorithm, which is designed to optimize a fitness function on the subset of the Boolean hypercube comprised of vectors of a fixed Hamming weight. The properties of this algorithm suit well for solving IM. The proposed algorithm is combined with the greedy heuristic for solving IM and TSS: the latter is used to construct initial solutions. We show that the described hybrid algorithms demonstrate significantly better performance compared to the computational scheme combining the greedy heuristic with the classic variant of (1 + 1)-EA. In the experiments, the proposed algorithms are applied to both real-world networks and the random networks constructed with respect to well-known models of random graphs. The results show that the new algorithms outperform the competition and are applicable to TSS and IM under DLTM for networks with tens of thousands of vertices.

## Introduction

Network science is a very fruitful and intensively developed area which features a lot of high quality publications. They can be broadly categorized into studies that analyze the properties related to network topology and papers that consider the properties of functions defined by networks. The former typically consider the so-called critical phenomena in networks and in this context they study various qualitative properties such as clustering, various consequences of the connectivity, such as a small world effect, degrees distributions, etc., as well as the models of random graphs that can be used to generate networks with desired properties. Since these questions do not constitute the main object of our study, let us cite in this context the reviews [1,2] and the book [3].

The scope of the present paper belongs to the second class of network science studies: the one that considers an existing network and analyzes various dynamic processes which occur inside said network. Thus the problems considered in the paper can be viewed as simple examples of agent-based modeling. Namely, we consider a network which models some collective: the network vertices interpret the members of collective (agents) and the edges interpret the connections between agents. With each network agent there is associated a special function that defines the degree of activity of this agent at each discrete time moment. The process in question models information dissemination in such a network, which can often be viewed as an activation process.

Among the central problems in the formulated context are the *Influence Maximization* problem (IM) and the related *Target Set Selection* problem (TSS). In just a few recent years hundreds of papers on these problems have been published, see e.g. [4–25]. In Sect 5 we will briefly review the main results achieved in these works. We would like to specifically note that the seminal paper [4] remains one of the main works on IM for more than 20 years. In that paper they performed the analysis of a number of key properties of activation processes in networks, and proved the combinatorial nature of both IM and TSS (e.g. showed that both are NP-hard). Also, in [4] it was demonstrated that under some models of information dissemination there exists an approximation algorithm for solving IM with constant multiplicative approximation factor of 1–*e*^−1^. This is the case for the so-called Linear Threshold Model and Independent Cascade Model. Both models are not deterministic in the base case, as the value of the objective function (influence in terms of [4]) is defined using some random variables. What is interesting, however, is that it is the non-deterministic nature of these models that is the reason for the existence of a 1–*e*^−1^-approximation algorithm for IM. In addition to that, [4] showed that under the deterministic variant of Linear Threshold Model (to which we further refer as to DLTM) IM already has tight arguments for inapproximability: specifically, if there exists an approximation algorithm with multiplicative constant factor for IM under DLTM, then *P* = *NP*.

Recall, that under DLTM the thresholds of vertices are fixed and therefore the influence spread follows a completely deterministic scenario. It means that its value is determined as the number of active vertices in the network at the moment when the activation process stops, or more precisely, when the activation algorithm does not make any inactive vertices active. DLTM looks to be quite reasonable since in applied scenarios the vertices’ thresholds usually have entirely practical interpretation and thus can be measured, similar to how it is done in the known threshold models of collective behavior [26].

Taking into account the high structural complexity of IM and TSS, the design of computational algorithms to solve these problems is a relevant direction of research. In the present paper, we propose several novel algorithms for finding an approximate solution for IM and TSS. In particular, we use as the basis of the proposed approaches the evolutionary algorithms which are applicable to solving optimization problems from a wide class, including the search for effective combinations of hyperparameters in Artificial Intelligence [27–29], etc. To justify the good accuracy of the proposed methods we find the exact solutions of network activation problems for networks of small size by reducing them to combinatorial problems, in particular to the Boolean satisfiability problem (SAT). The latter is solved using state-of-the-art SAT solvers which are actively used in combinatorial optimization and in the recent years find increasing use in the area of Explainable AI, see [30,31], etc.

The main novelty of our approach to IM and TSS solving is the combination of greedy algorithms with evolutionary algorithms and some additional heuristics. Specifically, the main contributions of our approach are the following:

1. We consider IM and TSS under DLTM in the context of the general pseudo-Boolean optimization problem which makes it possible to apply a wide variety of algorithms for their solving, including evolutionary algorithms;
2. We describe a new variant of (1 + 1)-Evolutionary algorithm ((1 + 1)-EA), which is designed to optimize a pseudo-Boolean fitness function on a subset of the Boolean hypercube of dimension *n* formed by the vectors of fixed size k:0<k<n and apply this algorithm to IM and TSS;
3. Using the networks of small size and reduction of TSS to the Boolean Satisfiability problem (SAT) we experimentally show that the metaheuristic algorithms for IM and TSS often make it possible to find solutions that are close to exact solutions;
4. We propose the hybrid strategy that combines the well-known greedy heuristic with evolutionary algorithms and show that it is applicable to solving IM and TSS under DLTM for real-world networks with tens of thousands of vertices.

Let us give a brief outline of our paper. The following Sect 1 is the preliminaries which contains the main notions, some auxiliary results, and also describes basic principles according to which evolutionary algorithms can be employed to optimize pseudo-Boolean fitness functions. Next Sect 2 contains the description of the (1 + 1)-Weighted Evolutionary Algorithm designed to optimize arbitrary pseudo-Boolean fitness functions over the subset of the Boolean hypercube formed by vectors of fixed Hamming weight. Further this algorithm together with several other evolutionary algorithms is used to solve IM and TSS. In Sect 3 we state IM and TSS under DLTM in form of pseudo-Boolean optimization problems, and describe a number of auxiliary heuristics used together with evolutionary algorithms when solving IM and TSS. The following Sect 4 presents the results of two series of computational experiments. In the first one we use networks of small size and reduction of TSS to SAT to construct exact solutions and show that the proposed metaheuristic algorithms often find either exact solutions or get very close to them. In the second series, we demonstrate the practical applicability of proposed algorithms to solving IM and TSS under DLTM for real-world networks from the SNAP repository [32] with thousands and tens of thousands of vertices. The paper is concluded with Sect 5 containing a brief review of related works and Sect 6 with conclusions and discussion of future work.

## 1 Preliminaries

It is convenient to consider DLTM in the context of the so-called *Synchronous Boolean Networks* (SBN), introduced by Stuart Kauffman in his seminal paper [33].

### 1.1 Synchronous Boolean networks and DLTM

Let us consider an arbitrary network as a directed labeled graph G=(V,A,L), where *V* is the set of vertices, *A* is the set of directed edges called *arcs*, and *L* is the set of labels associated with vertices and arcs. Assume that *G* does not have loops or multiple arcs. For an arbitrary vertex v∈V we say that the vertex u∈V is a *neighbor* of *v* if (u,v)∈A, i.e. if there is an arc in *G* that goes from *u* to *v*. The set of all neighbors of the vertex *v* is called the *neighborhood* of this vertex, which we denote as $U_{v}$.

Hereinafter, we suppose that each vertex from *G* is associated with a symbol from the set of size 2, which we denote by {0,1}. If a vertex *v* is associated with the number 1 then we say that *v* is *active*, otherwise *v* is assigned with the number 0 and is *inactive*. Apparently, it was S. Kauffman who proposed for the first time in his work [33] to associate with *G* the parameter *t* which takes the values in the set {0,1,2,…} and represents discrete time. The set of numbers from {0,1} connected to each network vertex at some time *t* forms a Boolean vector of length *n*, n=|V|. In this case, as it was shown by S. Kauffman, such Boolean vectors associated with the considered network at moments t∈{0,1,2,…} can be viewed as the states of a *Discrete Dynamical System* (DDS), in which the transitions between the states are specified by the deterministic rules, that do not depend on the specific time moment. S. Kauffman viewed the value from {0,1} associated with vertex *v* at moment *t* + 1, *t* ≥ 0 as the value of some Boolean function of arity $\left|U_{v}\right|$ which takes as an input the values assigned to vertices from $U_{v}$ at time moment *t*. Therefore, all coordinates of the Boolean vector of length *n* representing the network state at time moment t+1 are recomputed synchronously as the functions of the coordinates of the vector corresponding to the network state at time *t*. The described model is known as *Synchronous Boolean network* or *Kauffman network*. We will refer to the Boolean functions associated with network vertices the way outlined above as *weight functions*.

Since the rules that define the transitions between the states of Boolean network are deterministic, then the DDS defined this way, to which we further refer as DDS *G*, has a finite number of states. It means that, given the countable number of time states, such a DDS will traverse the same states multiple times. The situations of such a sort are defined as *attractors* or *cycles*. More strictly, let us represent the state of DDS *G* at an arbitrary time moment *t* by a Boolean vector of length *n* denoted as α(t). Consider the set of states α(t),α(t+1),…,α(t+j) such that *j* is the minimal possible number for which it holds that α(t)=α(t+j). In this case, we say that this set of states forms the *cycle* (attractor) of length *j*. If the states α(t),α(t+1) form a cycle of length 1, i.e. α(t)=α(t+1), then the state α(t) is called a *fixed point* of a considered DDS.

**Fig 1:**
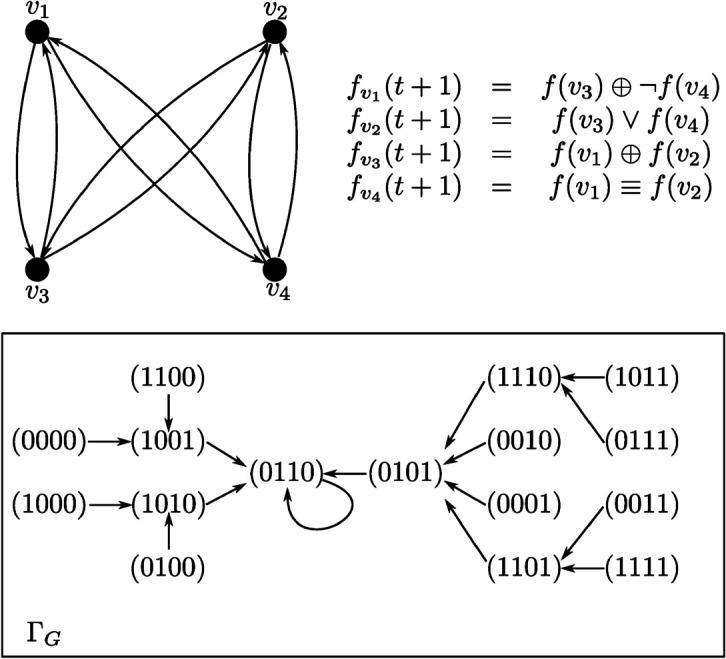
Example of a Synchronous Boolean Network with 4 vertices and its State Transition Graph.

Hereinafter, by {0,1}^*n*^ denote the set comprised of all binary vectors of length *n*. This set essentially is a *Boolean hypercube* of dimension *n*. Due to the deterministic nature of the weight functions of the vertices, we can view all 2^*n*^ different Boolean vectors from {0,1}^*n*^ as the states of DDS *G*. These states can be represented by the vertices of a directed graph called *State Transition Graph* (STG) and denoted by $\Gamma_{G}$. An arc (*a*,*b*) in $\Gamma_{G}$ interprets the transition from the state a=α(t) of DDS *G* to the state b=α(t+1). For two arbitrary states α(t),α(t+j), t≥0, j≥1, such that there exists a path from α(t) to α(t+j) in $\Gamma_{G}$ we will say that α(t+j) is *reachable* from α(t). All these notions are illustrated on Fig 1. For example, state (0110) is a fixed point. Coincidentally, it is reachable from all other states. On the other hand, state (0000) is not reachable from any other state.

It is possible to specify the weight functions of the vertices using different ways: by truth tables (as in [33]), by Boolean formulas (as in Fig 1) or by some predicates that express the relations between the corresponding elements (typically, in numeric form).

Further, we will mostly deal with DDS *G*, in which each arc (*u*,*v*) is assigned a non-negative rational number $B_{\left(u,v\right)}$. For correctness, assume that for each *v* at least one of the numbers $B_{\left(u,v\right)}$ is positive. Let us define the number $b_{\left(u,v\right)}$ as follows: $b_{\left(u,v\right)}=\frac{B_{\left(u,v\right)}}{\sum_{u\in U_{v}}B_{\left(u,v\right)}}$. Let us connect with an arbitrary vertex *v* some fixed number *θ*_*v*_ ∈ [0, 1], called *threshold* of *v*. Now, we define the weight function of vertex v∈V:

$$f_{v}(t\;+\;1)=\left\{ \begin{array}{cc} 1, & \sum_{u\in U_{v}}b_{\left(u,v\right)}\cdot f_{u}(t)\geq \theta_{v}\\ 0, & \text{otherwise} \end{array}\right.$$ (1)

The state of DDS *G* at the moment *t* = 0 is called *initial* and is specified separately. One can use any vector from {0,1}^*n*^ as an initial. A vertex *v* is called *active* at moment *t*, if $f_{v}(t)=1$, otherwise it is called *inactive* at this moment.

DDS *G* defined this way corresponds to the *Deterministic Linear Threshold Model* (DLTM) in the sense of [4].

### 1.2 IM and TSS w.r.t. DLTM

The following theorem is widely known and it follows from the fact that at time moment t+1 the number of active vertices in the network is greater or equal than the number of active vertices at moment *t*. The latter is, in turn, ensured by the properties of weight functions (1).

**Theorem 1.** *For an arbitrary DDS G defined above with weight functions of the kind (*1*) it holds that for any initial state α(0) there exists such t∈{0,1,2,…,n} that α(t), reachable from α(0) is a fixed point of DDS G.*

Hereinafter, for an arbitrary $\alpha (0)\in \{0,1{\}}^{n}$ we will be interested in the smallest *t* such that α(t) is a fixed point reachable from α(0). Let us denote the corresponding fixed point as $\overset{―}{\alpha }$. We will refer to the transition of DDS *G* from state α(0) to a fixed point $\overset{―}{\alpha }$ as to *activation process*.

**Definition 1.** *Consider an arbitrary DDS G w.r.t. DLTM defined above. For any vector $\alpha \in \{0,1{\}}^{n}$ define the influence σ(α) as the number of active vertices in G at fixed point $\overset{―}{\alpha }$ reachable from the initial state specified by *α*.*

For an arbitrary vector $\alpha \in \{0,1{\}}^{n}$ denote by $wt_{H}(\alpha )$ the Hamming weight [34] of the considered vector. For fixed k∈{1,…,n} denote by $\{0,1{\}}_{k}^{n}$ the set of all $\alpha \in \{0,1{\}}^{n}$ s.t. $wt_{H}(\alpha )=k$.

**Definition 2.** *Influence Maximization problem (IM) w.r.t. DLTM for specific value of parameter k consists in finding the following vector:*

$$\alpha^{*}=argmax_{\alpha \in \{0,1{\}}_{k}^{n}}\sigma (\alpha )$$ (2)

In other words, with each vector *α* from $\{0,1{\}}_{k}^{n}$ one associates the value σ(α) equal to the number of active vertices the network has in a fixed point reachable from *α*. We denote by $\alpha^{*}$ such a vector that for any $\alpha \in \{0,1{\}}_{k}^{n}$ it holds that $\sigma (\alpha^{*})\geq \sigma (\alpha )$.

In the context of IM the set of vertices which are active in initial state *α* is often referred to as a *target set*. Thus, any vector from {0,1}^*n*^ specifies some target set and vice versa. Taking this into account, we will use the term “target set" for an arbitrary $\alpha \in \{0,1{\}}^{n}$ considered as an initial state of DDS *G*.

The *Target Set Selection* (TSS) problem [4,35] is closely related to IM.

**Definition 3.** *The TSS w.r.t. DLTM for the predefined number R, 1≤R≤n consists in finding a target set *α* of minimal Hamming weight such that σ(α)≥R.*

Note, that while in IM one searches for a vector of particular weight *k* which maximizes the value of *σ* over $\{0,1{\}}_{k}^{n}$, in TSS one is looking for a vector *α* of minimal weight in the whole hypercube {0,1}^*n*^, such that σ(α)≥R.

### 1.3 Greedy heuristics for IM and TSS w.r.t. DLTM

As said above, there exist effective approximate algorithms for solving IM w.r.t. non-deterministic variant of linear threshold model (to which we further refer as LTM), such as the one considered in [4].

The weight functions in the framework of LTM are the same as functions (1) but the thresholds of vertices are randomly chosen. In more detail, we consider a probabilistic experiment in which the sample space is [0,1]^*n*^ (*n*-th Cartesian degree of the segment [0,1]). Each replica of [0,1] is connected to a separate vertex of network *G* and each [0,1] has a uniform distribution, according to which the threshold $\theta_{v}$ for the corresponding vertex v∈V is selected. For an arbitrary $\alpha \in \{0,1{\}}^{n}$ on [0,1]^*n*^ a random variable ξ(α) is defined, the value of which for a random set of thresholds from [0,1]^*n*^ is equal to the number of active vertices in $\overset{―}{\alpha }$ reached by DDS *G* from the initial state *α*. Influence $\tilde{\sigma }(\alpha )$ of specific $\alpha \in \{0,1{\}}^{n}$ is defined as the expected value of ξ(α):

$$\tilde{\sigma }(\alpha )=E[\xi (\alpha )]$$ (3)

For any k∈{1,…,n} IM w.r.t. the considered model consists in finding


$$\alpha^{*}=argmax_{\alpha \in \{0,1{\}}_{k}^{n}}\tilde{\sigma }(\alpha )$$

As it was shown in [4], the function (3) is submodular and hence the simple greedy algorithm from [36] can be used to solve this variant of IM, and it approximates the desired optimal solution with constant multiplicative factor equal to 1–*e*^−1^ in the following sense: if $\alpha^{\prime }$ is the solution found by the greedy algorithm then the following inequality holds: $\tilde{\sigma }(\alpha^{\prime })\geq (1-e^{-1})\cdot \tilde{\sigma }(\alpha^{*})$ (It should be noted that this is valid only under the assumption that the exact values of function (3) are given by some oracle).

On the other hand, according to [4], the function σ(·) is in general not submodular and cannot be approximated with any constant multiplicative factor under the assumption that P≠NP. Therefore, it is reasonable to apply various heuristics and metaheuristics for solving IM under DLTM.

Apparently, the first algorithm for solving IM and TSS specifically under DLTM is the greedy heuristic proposed in [37]. Let us give its brief description. This algorithm is quite similar to the greedy algorithm from [4]: that is, it starts with an empty set and at each subsequent step one vertex is added to the current target set for which the *influence measure* of this vertex is maximal. To define this measure, the concept of residual threshold of an arbitrary vertex is used, which we give below.

Consider an arbitrary state of DDS *G* given by a vector $\alpha \in \{0,1{\}}^{n}$. Let us associate the following value with *α* and an arbitrary vertex v∈V:

$$\Theta_{v}(\alpha )=\theta_{v}-\sum_{u\in U_{v}}b_{\left(u,v\right)}\cdot g_{u}(\alpha )$$ (4)

The function $g_{u}(\alpha )$ in (4) is defined as follows:


$$g_{u}(\alpha )=\left\{ \begin{array}{cc} 1, & if\;u\text{ is active in }\alpha \\ 0, & \text{otherwise} \end{array}\right.$$

**Definition 4.** *We will call the value given by the formula (*4*) a residual threshold of vertex v in state *α*.*

Obviously, if $\Theta_{v}(\alpha )\leq 0$, then *v* is active in state *α*, otherwise it is inactive in this state. Let us now describe the greedy heuristic which is a small modification of the algorithm proposed in [37]. Given the above, we will view an arbitrary target set as both a concrete set of vertices in the network *G* and as a Boolean vector of length *n*, where *n* is the number of vertices in *G*: the ones in such a vector mark vertices in target set under consideration.

Let us construct target sets of Hamming weight *k*, k∈{0,1,…,n}, using a strategy similar to the one employed in [4]. The values of *k* will correspond to the steps of the algorithm. At each step, the Hamming weight of the current target set is increased by 1 compared to the previous step. Denote the target set at step *k* by *T*_*k*_. For *k* = 0, we have $T_{k}=\emptyset$. To transition from *T*_*k*_ to $T_{k\;+\;1}$, we need to select some inactive vertex $v\in V⧵T_{k}$ and construct $T_{k\;+\;1}=T_{k}\cup \{v\}$. To choose such *v*, we compute the influence measure of vertices from $V⧵T_{k}$.

In more detail, for each vertex $v\in V⧵T_{k}$, we do the following:

1. Construct a *probing target set* ${\tilde{T}}_{k\;+\;1}=T_{k}\cup \{v\}$
2. Denote by $\tilde{\alpha }$ the state of DDS *G* corresponding to ${\tilde{T}}_{k\;+\;1}$; considering $\tilde{\alpha }$ as the initial state of DDS *G*, run activation process and obtain the fixed point $\overset{―}{\alpha }$;
3. Select the set of $A_{v}$ vertices that were inactive in state $\tilde{\alpha }$ but became active in state $\overset{―}{\alpha }$, and the set of $N_{v}$ vertices that remained inactive in state $\overset{―}{\alpha }$;
4. For each vertex $u\in N_{v}$ let us calculate the following value:
  $$\Delta \Theta_{u}=\Theta_{u}(\tilde{\alpha })-\Theta_{u}(\overset{―}{\alpha })$$ (5)
  Note that (5) is the value by which the residual threshold of vertex *u* decreases as we move from $\tilde{\alpha }$ to $\overset{―}{\alpha }$. Consider the following value:
  $$I_{v}=|A_{v}|\;+\;\sum_{u\in N_{v}}\frac{\Delta \Theta_{u}}{\theta_{u}}$$ (6)
  It is clear that (6) can be viewed as a measure of the *v*’s influence on the activation process: *intuitively* the larger the value of (6) is, the more *v* is involved in the process.
5. Let us go from *T*_*k*_ to $T_{k\;+\;1}=T_{k}\;\cup \;\{v^{*}\}$, where $v^{*}\in V\;⧵\;T_{k}$ is a vertex with the maximum value (6). For each k∈{0,1,…,n}, we will consider the Boolean vector that corresponds to the TS *T*_*k*_ constructed in the way described above as an approximation of $\alpha^{*}$ defined according to (2).

The pseudocode of the algorithm is presented at Algorithm 1.

**Algorithm 1. Greedy algorithm for solving IM/TSS.**



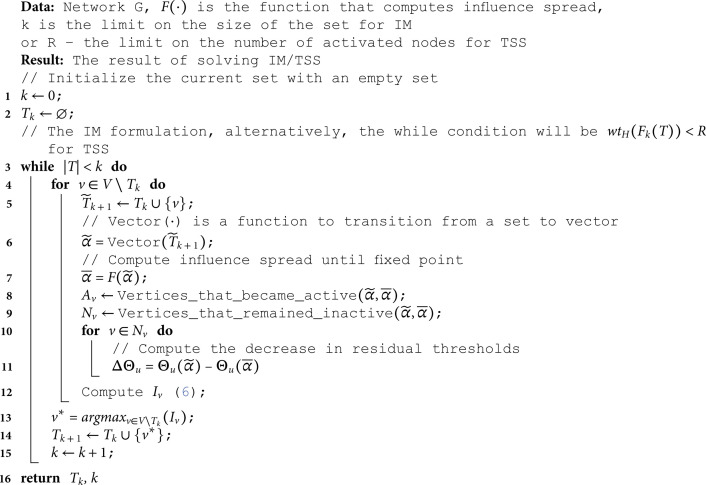



Note that the algorithm from [37] differs from the one described above only in that in [37] the activation process is run for some fixed number of time moments each time (specified by one of the algorithm parameters), without necessarily reaching a fixed point.

### 1.4 Finding exact solution to TSS using SAT solvers

It is easy to see that for relatively small networks one can find exact solutions of IM and TSS using exact combinatorial algorithms. In the present paper, for this purpose we employ the algorithms for solving the Boolean Satisfiability problem (SAT) [38], to which we refer as SAT solvers. Note that DDS *G* under DLTM defines a discrete function of the kind

$$\Phi_{G}:\{0,1{\}}^{n}\to \{0,1{\}}^{n}$$ (7)

where $\Phi_{G}$ receives as an input an arbitrary $\alpha \in \{0,1{\}}^{n}$, considered as the initial state of DDS *G*, and outputs a fixed point $\overset{―}{\alpha }$ reachable from *α*. Obviously, if the binary representation of numbers of the kind $\theta_{v}$ and $b_{\left(u,v\right)}$ do not depend on *n* or are bounded polynomially from *n*, then the function (7) is computable in polynomial time in *n*. In this case, due to the Cook-Levin theorem [39,40] there exists a procedure with the runtime polynomial in *n* that reduces IM and TSS under DLTM to SAT. Recall that SAT [38] is a problem in which one has to determine the satisfiability of an arbitrary Boolean formula ℱ, that is, to answer the question whether there exists a set of values of the variables in ℱ whose substitution into ℱ converts this formula to 1 (True). SAT is a classical NP-complete and NP-hard problem [41], therefore, under the assumption that P≠NP this problem cannot be solved in the general case in polynomial time. However, it is often the case that the SAT instances of large dimension can be solved in reasonable time with the help of modern SAT solvers [38]. This fact has stimulated the application of SAT solvers to an extremely wide class of combinatorial problems, which we will briefly discuss in Sect 5.

In the paper [42] the SAT solvers were applied to the study of the phenomena of conforming behavior in Kauffman networks. In essence, that paper solved TSS for DDS *G* under DLTM for functions of the kind (1), where the values $B_{\left(u,v\right)}$ were chosen from {0,1}. Unfortunately, this approach is applicable only to networks with at most a few hundred vertices and, therefore, can not be used to analyze real-world networks of practical interest. However, one feature of this approach is that it makes it possible to construct exact solutions of TSS for networks of small size, and therefore gives us an opportunity to understand how far the solutions obtained by metaheuristic algorithms for these networks are from the exact ones. This will give us additional arguments in favor of the accuracy of the metaheuristics we use.

## 2 Evolutionary optimization

The main novelty of this paper is the use of metaheuristic optimization algorithms, specifically evolutionary algorithms, to solve IM and TSS. We will use such algorithms to optimize pseudo-Boolean functions which are naturally connected with IM and TSS.

Recall that a pseudo-Boolean function [43] is any function of the following form:

$$F:\{0,1{\}}^{n}\to \mathbb{R}$$ (8)

In many real-world problems, there is no information about the analytic properties of functions of the form (8), and in such cases it is reasonable to apply metaheuristic algorithms to find the optimum of *F* over the hypercube {0,1}^*n*^. In the role of such algorithms, one can use, for example, various local search strategies [44], but in our paper we employ the algorithms of evolutionary optimization [45] because they often show good results in application to similar problems from related areas.

Let us briefly present the main notions and constructions used below. The simplest evolutionary algorithm is (1 + 1)-EA [46]. It is based on the notion of *random mutation*: with an arbitrary Boolean vector $\alpha \in \{0,1{\}}^{n}$: $\alpha =(\alpha_{1},\ldots ,\alpha_{n})$ associate a sequence of independent Bernoulli trials [47] of length *n*: $\beta_{1},\ldots ,\beta_{n}$ with a fixed probability of success *p* (called *mutation rate*) in each trial. For each i∈{1,…,n}: if $\beta_{i}=0$, then the $\alpha_{i}$ coordinate of vector *α* remains unchanged; if $\beta_{i}=1$, then the $\alpha_{i}$ coordinate is flipped, that is, 0→1 or 1→0. This process yields the vector $\alpha^{\prime }$; $\alpha^{\prime }$ is said to be the result of a random mutation of *α* with mutation rate *p*. If *α* is the current vector and $\alpha^{\prime }$ is the result of its random mutation such that $F(\alpha^{\prime })>F(\alpha )$ (assuming that we consider the maximization problem for (8)) then $\alpha^{\prime }$ becomes the new current vector; otherwise *α* remains as the current vector. Random mutations are applied to the current vector until some termination criterion is met (e.g., a limit on the number of mutations is exceeded).

Despite its simple nature, (1 + 1)-EA has many important theoretical properties. For example, it is easy to see that when $p=\frac{1}{n}$, the expected value of random variable $d_{H}(\alpha ,\alpha^{\prime })$ (Hamming distance between *α* and $\alpha^{\prime }$) is 1. This property is very important because it means that, on average, (1 + 1)-EA behaves like a simple Hill Climbing (HC) algorithm [48] and thus can adjust to the landscape of function *F*, efficiently climbing to local extremums. On the other hand, unlike HC, (1 + 1)-EA is able to move with non-zero probability from an arbitrary point *α* to a point $\alpha^{*}$ where the function (8) has a global extremum over {0,1}^*n*^.

A number of important theoretical properties of (1 + 1)-EA were presented in the seminal paper [49]. In particular, that paper introduced the notion of complexity for this algorithm and showed that when *p* = 1/*n* in the general case, the algorithm is extremely inefficient, since its complexity interpreted as the expected value of the number of random mutations required to reach $\alpha^{*}$ for a number of special functions is $n^{\varepsilon n}$ for some constant ε∈[0,1]. Thus, in the sense of this measure, (1 + 1)-EA has worse complexity even compared to the simple random walk in which we randomly choose *α* w.r.t. uniform distribution on {0,1}^*n*^. However, this result is a typical example of the worst case scenario whereas in application to many combinatorial problems (1 + 1)-EA is surprisingly effective and often performs better than more sophisticated algorithms. One of the reasons for this is the small expected number of flipped bits in one random mutation, which makes (1 + 1)-EA on average similar to HC [50]. Another advantage of (1 + 1)-EA is that it is easy implement and has a high speed of operation.

In several papers there have been proposed alternative variants of (1 + 1)-EA, the goal of which was to reduce the upper bound on its complexity. The (1 + 1)-Fast Evolutionary Algorithm proposed in [51] is among the best approaches of such a kind. It employs a parameter *β* and is usually denoted as (1 + 1)-FEA_β_. The main idea of that algorithm is to adjust the mutation rate during its work. In more detail, before mutating each $\alpha \in \{0,1{\}}^{n}$ one observes the value of a special random variable *δ* with the spectrum $S_{\delta }=\{1,2,\ldots ,\frac{n}{2}\}$. The distribution of *δ* (also called “power-law distribution $D_{n/2}^{\beta }$” [51]) is defined in the following manner:

$$\mathrm{Pr}\{\delta =k,k\in S_{\delta }\}={\left(C_{n/2}^{\beta }\right)}^{-1}\cdot \frac{1}{k^{\beta }}$$ (9)

In the Eq 9 *β*, β>1 is the algorithm’s parameter and $C_{n/2}^{\beta }$ is the so-called *normalizing constant* used to normalize probabilities: $C_{n/2}^{\beta }=\sum_{i=1}^{n/2}i^{-\beta }$. Then, a single mutation in (1 + 1)-FEA_β_ is performed as follows:

1. Generate a value of δ∈{1,…,n/2} according to the distribution $D_{n/2}^{\beta }$
2. Apply the standard mutation operator with p=δ/n to $\alpha \in \{0,1{\}}^{n}$.

The upper bound on the complexity of (1 + 1)- FEA_β_ is $O(C_{n/2}^{\beta }\;\cdot \;n^{\beta }\;\cdot \;2^{n})$ which is asymptotically significantly better than that for (1 + 1)-EA. Meanwhile, the expected value of flipped bits of (1 +  1)- FEA_β_ is a constant independent of *n* only when β>2. For example in the case β=3 we have $E[d_{H}(\alpha ,\alpha^{\prime })]=1.3685\ldots$ (see [51]).

Genetic algorithms (GA) [45] are often used to solve pseudo-Boolean optimization problems, and they can also be applied to IM and TSS. In this paper, we employ the following variant of GA.

Suppose that there is a set of Boolean vectors from {0,1}^*n*^, called *population*. In particular, we will talk about the current population and use the notation $P_{curr}=\{\alpha^{1},\ldots ,\alpha^{s}\}$. The vectors $\alpha^{j}$, j∈{1,…,s} are referred to as *individuals*. Let us set the probability distribution $D_{curr}=\{p_{1},\ldots ,p_{s}\}$ on *P*_*curr*_ in accordance with the following rules (assuming that we aim to maximize (8) over {0,1}^*n*^):


$$p_{i}=\frac{F(\alpha^{i})}{\sum_{j=1}^{s}F(\alpha^{j})},i\in \{1,\ldots ,s\}$$

Next, we select *l* individuals with the best values of function (8) from *P*_*curr*_ and add these individuals into new population *P*_*new*_. Then, in accordance with the distribution *D*_*curr*_, we select *g* random individuals from *P*_*curr*_ and apply to each of them random mutation with mutation rate *p* = 1/*n* or the mutation variant from [51]. The obtained individuals are added to the new population *P*_*new*_. Finally, we construct *h* pairs of randomly chosen vectors from *P*_*curr*_ w.r.t. distribution *D*_*curr*_ and apply to each pair some variant of the *crossover* operator [45] (in the experiments we used standard two-point crossover) and add the resulting vectors to *P*_*new*_. The values of *l*,*g*,*h* are selected so that the relation l+g+2h=s is satisfied. After this the constructed population *P*_*new*_ becomes *P*_*curr*_.

### 2.1 A new variant of (1 + 1)-EA

In this section we describe the algorithm that seeks an extremum of an arbitrary function of the kind (8) over $\{0,1{\}}_{k}^{n}$. We will later apply this algorithm to solving IM and TSS.

Consider arbitrary k,n∈N: 0<*k*<*n* and the set $\{0,1{\}}_{k}^{n}$ formed by all vectors from the hypercube {0,1}^*n*^ with Hamming weight *k*. Assume that we have an optimization problem for some pseudo-Boolean function of the kind (8) over $\{0,1{\}}_{k}^{n}$. Our closest goal is to construct an algorithm for solving this problem which inherits the basic properties of (1 + 1)-EA. The main idea of this algorithm is to apply the standard (1 + 1) mutation to some part of $\alpha \in \{0,1{\}}_{k}^{n}$, and then use the so-called *correcting transformation* on the other part. Let us present the details below.

By |α| let us denote the length of vector *α*. For an arbitrary $\alpha \in \{0,1{\}}_{k}^{n}$ we split it into two parts $\gamma_{1},\gamma_{0}:\alpha =(\gamma_{1},\gamma_{0})$. Vectors $\gamma_{1}$ and $\gamma_{0}$ are formed by the coordinates of *α* which are equal to 1 and 0, respectively. We assume that $|\gamma_{1}|=k$ and $|\gamma_{0}|=n\;-\;k$. Next, we define the transformation of *α* as the transformation of vectors $\gamma_{1},\gamma_{0}$.

We will use the following notation: $\gamma^{\prime }\in \{\gamma_{1},\gamma_{0}\}:|\gamma^{\prime }|=\min\{|\gamma_{1}|,|\gamma_{0}|\}$ and $\gamma^{\prime \prime }=\{\gamma_{1},\gamma_{0}\}\;⧵\;\{\gamma^{\prime }\}$. Apply the standard random mutation operator with rate $p=1/|\gamma^{\prime }|$ to $\gamma^{\prime }$. If no bits of $\gamma^{\prime }$ were flipped during the mutation then $\alpha^{\prime }=\alpha$. Now, suppose that *r* bits in $\gamma^{\prime }$ were flipped, *r*>0. Let us fix the coordinates of flipped bits in $\gamma^{\prime }$. We need to apply to $\gamma^{\prime \prime }$ such a transformation that the Hamming weight of the result of mutation of *α* will be equal to that of *α*. For this purpose, we choose *r* random coordinates in $\gamma^{\prime \prime }$ using the standard sampling without replacement [47], and flip the bits with these coordinates in *α*. Denote the resulting vector by $\tilde{\alpha }$. It is clear that $\tilde{\alpha }\in \{0,1{\}}_{k}^{n}\;$. We denote the operator that maps $\{0,1{\}}_{k}^{n}$ onto itself as $\mu_{k}^{n}:\mu_{k}^{n}(\alpha )=\tilde{\alpha }$. For convenience, we will also denote the images of vectors $\gamma^{\prime }$ and $\gamma^{\prime \prime }$ in $\tilde{\alpha }$ w.r.t. $\mu_{k}^{n}$ as ${\tilde{\gamma }}^{\prime }$ and ${\tilde{\gamma }}^{\prime \prime }$. Let us refer to the $\mu_{k}^{n}$ operator as *k*-*weight random mutation*.

Note that we can employ the operator $\mu_{k}^{n}$ to perform transitions between vectors from $\{0,1{\}}_{k}^{n}$ similar to how the standard random mutation operator in (1 + 1)-EA is employed: if *α* is the current vector from $\{0,1{\}}_{k}^{n}$, and $\alpha^{\prime }=\mu_{k}^{n}(\alpha )$, then $\alpha^{\prime }$ becomes current only if $F(\alpha^{\prime })>F(\alpha )$ (assuming that we aim to maximize *F*). The pseudocode of this algorithm is presented in Algorithm 2. Further we will refer to it as (1 + 1)-WEA (from ‘Weighted Evolutionary Algorithm’). Let us establish the main properties of the algorithm.

**Algorithm 2. The pseudocode for (1 + 1)-WEA.**



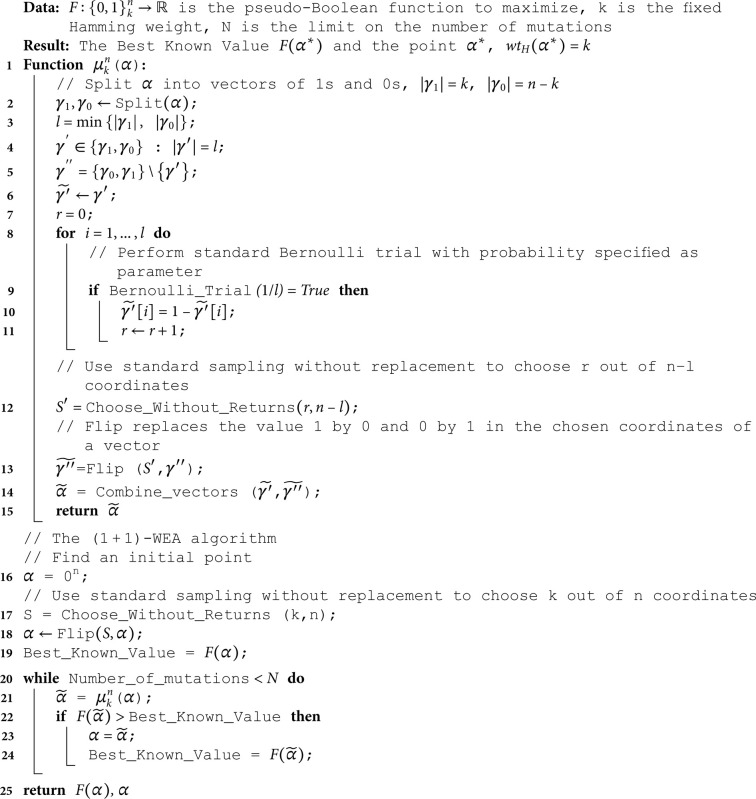



**Proposition 1.** *For any two vectors $\alpha ,\tilde{\alpha }\in \{0,1{\}}_{k}^{n}$ there exists a transition of the kind $\mu_{k}^{n}(\alpha )=\tilde{\alpha }$.*

*Proof*: Consider two arbitrary vectors $\alpha ,\tilde{\alpha }\in \{0,1{\}}_{k}^{n}$ and outline the part $\gamma^{\prime }$ in *α*. Without loss of generality, assume that vector $\gamma^{\prime }$ is formed by ones and $|\gamma^{\prime }|=k$. Assume that the coordinates of $\gamma^{\prime }$ have the numbers $i_{1},\ldots ,i_{k}$, and the coordinates of $\gamma^{\prime \prime }$ have the numbers $j_{1},\ldots ,j_{n-k}$ with respect to the order (of coordinates) common for both *α* and $\tilde{\alpha }$. Let ${\tilde{\lambda }}^{\prime },{\tilde{\lambda }}^{\prime \prime }$ be the vectors formed by the coordinates with numbers $i_{1},\ldots ,i_{k}$ in vector $\tilde{\alpha }$ and numbers $j_{1},\ldots ,j_{n-k}$ in vector $\tilde{\alpha }$ respectively. Note that if $d_{H}(\gamma^{\prime },{\tilde{\lambda }}^{\prime })=0$, then $d_{H}(\gamma^{\prime \prime },{\tilde{\lambda }}^{\prime \prime })=0$, thus $\alpha =\tilde{\alpha }$. Therefore, in this case the transition $\alpha \to \tilde{\alpha }$ is realized by means of the event in which during the (1 + 1)-random mutation of $\gamma^{\prime }$ there are no flipped bits. This event has non-zero probability and, therefore, can be realized. Suppose that $d_{H}(\gamma^{\prime },{\tilde{\lambda }}^{\prime })=t$, 1≤t≤k. It means that vector ${\tilde{\lambda }}^{\prime }$ is formed by *t* zeroes and *k*–*t* ones. But in this case, the vector ${\tilde{\lambda }}^{\prime \prime }$ must contain *t* ones and, accordingly, *n*–*k*–*t* zeros. Then it is easy to see that the transition $\alpha \to \tilde{\alpha }$ is also possible (and consequently has non-zero probability) thanks to the use of the $\mu_{k}^{n}$ operator: ${\tilde{\gamma }}^{\prime }={\tilde{\lambda }}^{\prime }$; ${\tilde{\gamma }}^{\prime \prime }={\tilde{\lambda }}^{\prime \prime }$. Due to the fact that we can arbitrarily choose vectors $\alpha ,\tilde{\alpha }$ from $\{0,1{\}}_{k}^{n}$ the proposition is proved. □

As we already noted above, it is important that the vector which is the random mutation of *α* is as close to *α* as possible Hamming distance-wise, since it enables the algorithm to ‘sense’ the landscape of a plot of a considered function. Note that for an arbitrary pair of vectors $\alpha ,\tilde{\alpha }\in \{0,1{\}}_{k}^{n}$: $\alpha \neq \tilde{\alpha }$ we have $d_{H}(\alpha ,\tilde{\alpha })\geq 2$. Now, let us establish the following property of the described algorithm.

**Proposition 2.** *For $\alpha ,\tilde{\alpha }\in \{0,1{\}}_{k}^{n}$: $\tilde{\alpha }=\mu_{k}^{n}(\alpha )$ it holds that $E[d_{H}(\alpha ,\tilde{\alpha })]=2$.*

*Proof*: Assume that the application of $\mu_{k}^{k}$ to a part $\gamma^{\prime }$ of vector *α* resulted in the flipping of *r* bits. But in this case in $\gamma^{\prime \prime }$ exactly *r* bits will also be flipped (chosen randomly). Then it holds that $E[d_{H}(\alpha ,\tilde{\alpha })]=E[2\;\cdot \;d_{H}(\gamma^{\prime },{\tilde{\gamma }}^{\prime })]=2\;\cdot \;E[d_{H}(\gamma^{\prime },{\tilde{\gamma }}^{\prime })]$. Since the expected value of the number of flipped bits in $\gamma^{\prime }$ w.r.t. mutation rate $p=1/|\gamma^{\prime }|$ is 1 (see above), we have that $E[d_{H}(\alpha ,\tilde{\alpha })]=2$. Thus the proposition is proved. □

Now let us construct an upper bound on the complexity of the described algorithm. We will use the reasoning similar to that from [49]. Remind that in [49] they proposed the idea to estimate the complexity of (1 + 1)-EA via the expected value of a random variable which is the number of random mutations performed by (1 + 1)-EA until it reaches a global extremum of the considered function. We will denote this expectation for specific $\alpha \in \{0,1{\}}^{n}$ as $E[\alpha \to \alpha^{*}]$. Let *P* be the probability of success of the event $\left\{\alpha \to \alpha^{*}\right\}$. Then due to the properties of the geometric distribution [47] we have that the considered expectation is $E[\alpha \to \alpha^{*}]=1/P$.

In [49] an apparatus was presented which makes it possible to construct upper bounds for values of the kind $E[\alpha \to \alpha^{*}]$ keeping in mind an arbitrary vector *α*. As it was showed in [49] the value *P* reaches its minimum for *α* such that $d_{H}(\alpha ,\alpha^{*})=n$ and in this case we have *P* = *p*^*n*^ where *p* is a mutation rate. If, for example, $p=\frac{1}{n}$, then we have *P* = *n*^−*n*^ and, consequently, the following upper bound on the complexity of (1 + 1)-EA holds: $E[\alpha \to \alpha^{*}]\leq n^{n}$. As mentioned above, for some specific functions (see [49]) it was demonstrated that bounds of the kind $n^{\varepsilon \;\cdot \;n}$ can be achieved for some constant ε∈[0,1]. However, in our case, we know nothing about the nature of the considered functions and, consequently, we need to work under the assumption that this function is a black box and construct for (1 + 1)-WEA only the upper bounds similar to ones for the classical variant of (1 + 1)-EA. The corresponding results are presented by the following theorem.

**Theorem 2.** *Suppose, that (1 + 1)-WEA is applied to maximization of an arbitrary pseudo-Boolean function over $\{0,1{\}}_{k}^{n}$, 0<k<n, and let l=min{k,n−k}. Then for some $n_{0}\in \mathbb{N}$ and for all $n\geq n_{0}$ the following upper bound holds:*


$$E[\alpha \to \alpha^{*}]\leq 2\;\cdot \;{\left(\frac{l\;\cdot \;(n-l)}{e^{1\;+\;\frac{1}{n-l}}}\right)}^{l}$$

*Proof*: Fix an order on the set of coordinates of all vectors from {0,1}^*n*^. Consider the operator $\mu_{k}^{n}:\{0,1{\}}_{k}^{n}\to \{0,1{\}}_{k}^{n}$. Let us construct vectors $\gamma^{\prime }$ and $\gamma^{\prime \prime }$ and assume, that $|\gamma^{\prime }|=l$, $|\gamma^{\prime \prime }|=n\;-\;l$. From the definition of $\mu_{k}^{n}$ it follows that l≤n/2. The vector $\gamma^{\prime }$ undergoes the standard (1 + 1)-EA mutation with the rate *p* = 1/*l*. Let $\alpha^{*}\in \{0,1{\}}_{k}^{n}$ be the vector on which the considered (arbitrary) fitness function achieves a global extremum in $\{0,1{\}}_{k}^{n}$. Denote by $\gamma^{\prime *}$ the vector formed by the coordinates in $\alpha^{*}$ with the same numbers (w.r.t. the order fixed above) as in $\gamma^{\prime }$. We want to estimate the probability that after the application of $\mu_{k}^{n}$ to *α* the following transition happens: $\gamma^{\prime }\to \gamma^{\prime *}$. By using the reasoning similar to that in [49], we have that:

$$\mathrm{Pr}\left\{\gamma^{\prime }\to \gamma^{\prime *}\right\}={\left(\frac{1}{l}\right)}^{d_{H}\left(\gamma^{\prime },\gamma^{\prime *}\right)}\;\cdot \;{\left(1-\frac{1}{l}\right)}^{l-d_{H}\left(\gamma^{\prime },\gamma^{\prime *}\right)}$$ (10)

Suppose, that during the transition $\mu_{k}^{n}(\alpha )=\alpha^{*}$ there were *r* (r≤l) mutated bits in $\gamma^{\prime }$, and the result of transition was $\gamma^{\prime }\to \gamma^{\prime *}$. Thus, $r=d_{H}(\gamma^{\prime },\gamma^{\prime *})$. Now, consider the corresponding correcting transformation of $\gamma^{\prime \prime }$. Under the assumption that the transition is $\gamma^{\prime \prime }\to \gamma^{{*}^{\prime \prime }}$ we can conclude, that $\gamma^{\prime \prime }$ and $\gamma^{{*}^{\prime \prime }}$ must differ in *r* coordinates, and these are the exact coordinates that must be flipped during the application of $\mu_{k}^{n}$ to $\gamma^{\prime \prime }$. Thus, we need to correctly choose *r* coordinates out of *n*–*l*, using the sampling without replacement. It is a well-known fact (see e.g. [47]) that if the order of chosen coordinates matters, then the corresponding number of possible alternative choices is expressed by the following falling factorial: $\left(n\;-\;l{)}_{r}=(n\;-\;l)\;\cdot \;\ldots \;\cdot \;(n\;-\;l\;-\;r\;+\;1\right)$. But in our case, the order of chosen coordinates does not matter because *n*–*l* considered coordinates are either all zeroes or all ones. Thus, the number of possible alternatives of the choice is the following binomial coefficient: (n−lr). Then, the probability of the transition $\gamma^{\prime \prime }\to \gamma^{{*}^{\prime \prime }}$ looks as follows:

$$\mathrm{Pr}\left\{\gamma^{\prime \prime }\to \gamma^{{*}^{\prime \prime }}\right\}=\frac{r!}{(n-l{)}_{r}}$$ (11)

Note, that when *r* = 0 the vector *α* will not change and the probability of this event is ${\left(1-\frac{1}{l}\right)}^{l}\approx e^{-1}$. Hereinafter, we assume that r≥1. Taking into account (10) and (11), for a fixed r:1≤r≤l≤n/2  we have, that:


$$\mathrm{Pr}\left\{\alpha \to \alpha^{*}\right\}=\frac{r!}{l^{r}\;\cdot \;(n-l{)}_{r}}\;\cdot \;{\left(1-\frac{1}{l}\right)}^{l-r}$$

Let us make use of the fact, that ${\left(1-\frac{1}{l}\right)}^{l-r}={\left(1-\frac{1}{l}\right)}^{l\;\cdot \;(1-\frac{r}{l})}\approx e^{-(1-\frac{r}{l})}$. Then, when 1≤r≤l we have $e^{-1}<e^{-\left(1-\frac{r}{l}\right)}\leq 1$. Therefore,

$$\mathrm{Pr}\left\{\alpha \to \alpha^{*}\right\}\geq \frac{r!}{e\;\cdot \;l^{r}\;\cdot \;(n-l{)}_{r}}$$ (12)

From (12), using the reasoning analogous to [49], we have an expression for the expected value of the number of independent mutations that need to happen for the transition $\alpha \to \alpha^{*}$ to occur:

$$E\left[\alpha \to \alpha^{*}\right]\leq \frac{e}{r!}\;\cdot \;l^{r}\;\cdot \;(n-l{)}_{r}$$ (13)

To construct a more explicit estimation for $E\left[\alpha \to \alpha^{*}\right]$ let us introduce the notation $\Xi (n,l,r)=\frac{e}{r!}\;\cdot \;l^{r}\;\cdot \;(n\;-\;l{)}_{r}$ and consider the value:$\;\frac{1}{{\left(n-l\right)}^{r}}\Xi (n,l,r)$. Then, directly from (13) the following holds:


$$\frac{1}{(n-l{)}^{r}}\Xi (n,l,r)=\frac{e}{r!}\;\cdot \;l^{r}\;\cdot \;1\;\cdot \;\left(1-\frac{1}{n-l}\right)\;\cdot \;\ldots \;\cdot \;\left(1-\frac{r-1}{n-l}\right)$$

Taking into account, that r≤l≤n/2, for all r≥2 we have:

$$\frac{1}{(n-l{)}^{r}}\Xi (n,l,r)\leq \frac{e}{r!}\;\cdot \;l^{r}\;\cdot \;{\left(1-\frac{1}{n-l}\right)}^{r-1}=\frac{e{\left(1-\frac{1}{n-l}\right)}^{-1}}{r!}\;\cdot \;l^{r}\;\cdot \;{\left(1-\frac{1}{n-l}\right)}^{\frac{r(n-l)}{n-l}}$$ (14)

Note, that ${\left(1-\frac{1}{n-l}\right)}^{n-l}$ tends to *e*^−1^ with the increase of *n*. Then, keeping in mind that for all *r* > 6 it holds that $r!>e^{r\;+\;1}$ we can rewrite (14) as follows:

$$\begin{array}{cc} \frac{1}{(n-l{)}^{r}}\Xi (n,l,r) & \leq {\left(1-\frac{1}{n-l}\right)}^{-1}\;\cdot \;\frac{1}{e^{r}}\;\cdot \;l^{r}\;\cdot \;{\left(\frac{1}{e^{\frac{1}{n-l}}}\right)}^{r}=\\ & ={\left(1-\frac{1}{n-l}\right)}^{-1}\;\cdot \;{\left(\frac{l}{e^{1\;+\;\frac{1}{n-l}}}\right)}^{r} \end{array}$$ (15)

Since $l\leq \frac{n}{2}$, then $1<{\left(1-\frac{1}{n-l}\right)}^{-1}\leq 2$ for all n:n≥4. Also, taking into account (13) we can derive from (15) that:

$$E\left[\alpha \to \alpha^{*}\right]\leq 2\;\cdot \;{\left(\frac{l\;\cdot \;(n-l)}{e^{1\;+\;\frac{1}{n-l}}}\right)}^{r}$$ (16)

Let us note, that the value in the right part of (16) is increasing with the increase of *r*. However, since r≤l it is clear that the validity of the theorem follows from (16). □

## 3 IM and TSS as pseudo-Boolean optimization problems

In this section we will give formal definitions of IM and TSS in form of maximization problems for pseudo-Boolean functions of the kind (8). To solve them we will employ the concept in which the solving itself is split in two stages:

1. The stage at which we construct an initial solution using a Greedy Algorithm (GrA);
2. The stage at which we improve the solution found at stage 1 using evolutionary optimization.

In the context of IM we consider DDS *G*, |V|=n with weight functions of the kind (1) and fixed thresholds (DLTM). For each k∈{1,…,n} let us focus on the following function:

$$F_{k}:\{0,1{\}}_{k}^{n}\to \{1,\ldots ,n\}$$ (17)

For an arbitrary $\alpha \in \{0,1{\}}_{k}^{n}$ we will suppose that $F_{k}(\alpha )=\sigma (\alpha )$, where σ(·) is the influence computed w.r.t. Definition 2.

Using GrA let us find some $\alpha^{0}\in \{0,1{\}}_{k}^{n}$ which will serve as an initial approximation of $\alpha^{*}$, where $\alpha^{*}$ is defined by (2). Next, we use $\alpha^{0}$ as a starting current point and apply *k*-weight mutation operator $\mu_{k}^{n}$ to $\alpha^{0}$ and the following current vectors, aiming to maximize (17) until some termination criterion is met. The resulting vector ${\tilde{\alpha }}^{*}$ is the approximate solution of IM for network *G* over $\{0,1{\}}_{k}^{n}$. Let us additionally note, that to find $\alpha^{0}\in \{0,1{\}}_{k}^{n}$, k≥1 the greedy algorithm uses the approximate solutions of IM over the sets of the kind $\{0,1{\}}_{i}^{n}$, i∈{0,…,k−1}.

Next, consider the TSS problem. The general scheme for solving TSS employed further is as follows. On the first stage, similar to IM we construct an approximate solution $\alpha^{0}$ using GrA. Thus, the vector $\alpha^{0}$ is an approximate solution for IM for the smallest *k* such that the number of active vertices in the state $\overset{―}{\alpha }$ reachable from $\alpha^{0}$ is at least *R*.

Once we found $\alpha^{0}$ in $\{0,1{\}}_{k}^{n}$ we make use of the two following strategies for solving TSS. The first strategy implies launching (1 + 1)-EA, (1 + 1)-FEA [51] or GA. Each algorithm is tasked with finding a vector of minimal Hamming weight such that starting from this vector, the DDS *G* transitions to a fixed point $\overset{―}{\alpha }$ in which the number of active vertices is at least *R*.

The second strategy consists in the following steps:

1. Start from the current dimension *k* such that $\alpha^{0}\in \{0,1{\}}_{k}^{n}$ and use only operator $\mu_{k}^{n}$ on $\{0,1{\}}_{k}^{n}$ to find some approximate solution of IM denoted by ${\tilde{\alpha }}_{k}^{*}\in \{0,1{\}}_{k}^{n}$;
2. Transition from ${\tilde{\alpha }}_{k}^{*}$ to some vector $\alpha_{k-1}^{0}\in \{0,1{\}}_{k-1}^{n}$ using the heuristics described below;
3. Starting from $\alpha_{k-1}^{0}$ apply operator $\mu_{k-1}^{n}$ and try to find such ${\tilde{\alpha }}_{k-1}^{*}\in \{0,1{\}}_{k-1}^{n}$ that the number of active vertices in fixed point $\overset{―}{\alpha }$ reached from ${\tilde{\alpha }}_{k-1}^{*}$ is at least *R*. In the case of success, decrease the current dimension by 1 and go to step 2. Otherwise, output the current best known vector ${\tilde{\alpha }}^{*}$ as an approximate solution of TSS.

To transit from ${\tilde{\alpha }}_{k}^{*}$ to $\alpha_{k-1}^{0}$ on step 2 we use several heuristics. Since for each value of *k* we will perform the same actions, let us without the loss of generality use the notation ${\tilde{\alpha }}^{*}$ instead of ${\tilde{\alpha }}_{k}^{*}$. An arbitrary TS defined by vector $\alpha \in \{0,1{\}}^{n}$ we will denote as T(α).

The first and the simplest heuristic, to which we further refer as to (1 + 1)-WEA_v1 consists in removing from the set $T({\tilde{\alpha }}^{*})$ the vertex with the smallest number of outgoing arcs.

In the second heuristic denoted as (1 + 1)-WEA_v2 for each vertex $v\in T({\tilde{\alpha }}^{*})$ we compute the so-called *activation potential*:

$$\pi (v)=\sum_{u:(v,u)\in A}\frac{b_{\left(v,u\right)}}{\theta_{u}}$$ (18)

Then we remove from $T({\tilde{\alpha }}^{*})$ the vertex with minimal value of π(v).

We also employed a heuristic which is close in spirit to the greedy algorithm described in Sect 1.3. Consider ${\tilde{\alpha }}^{*}$ and let $\overset{―}{\alpha }$ be the fixed point reached by DDS *G* when it starts from the initial state ${\tilde{\alpha }}^{*}$. For each vertex $v\in T({\tilde{\alpha }}^{*})$ denote by ${\tilde{\alpha }}_{v}^{*}$ the vector which specifies the target set $T({\tilde{\alpha }}^{*})\backslash \{v\}$ and let ${\overset{―}{\alpha }}_{v}$ be a fixed point reached by DDS *G* from state ${\tilde{\alpha }}_{v}^{*}$. Next we find the following vertex

$$v^{\prime }=argmin_{v\in T({\tilde{\alpha }}^{*})}({wt}_{H}(\overset{―}{\alpha })-wt_{H}({\overset{―}{\alpha }}_{v}))$$ (19)

In the role of $\alpha_{k-1}^{0}$ we pick the vector that specifies target set $T({\tilde{\alpha }}^{*})\backslash \{v^{\prime }\}$. However in practice this step is quite computationally intensive due to the necessity to check all vertices in $T({\tilde{\alpha }}^{*})$. That is why we sort all vertices from $T({\tilde{\alpha }}^{*})$ in an ascending order of value (18), calculate the difference $wt_{H}(\overset{―}{\alpha })\;-\;wt_{H}({\overset{―}{\alpha }}_{v})$ only for the first *Q* vertices from this ordered set, and choose a vertex with minimal value of this difference. We denote the described heuristic as (1 + 1)-WEA_v3. In the next section, we present the results that demonstrate the behavior of the described heuristic for different values of parameter *Q*. The pseudocode of the proposed strategy for solving TSS by iteratively decreasing the value of *k* using the heuristic variant v3 is presented at Algorithm 3.

**Algorithm 3. The hybrid algorithm with v3 heuristic.**



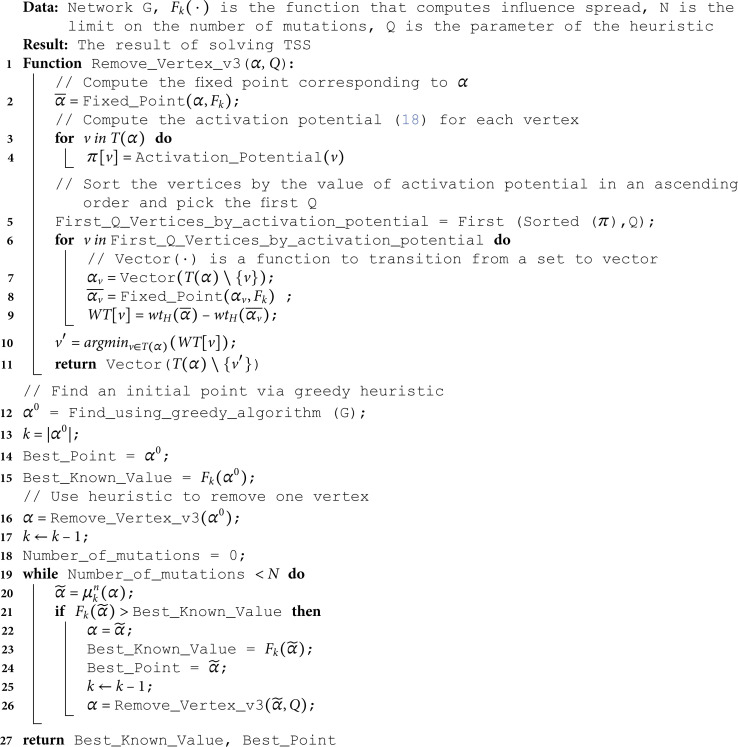



## 4 Computational experiments

We performed several series of experiments to assess the algorithms proposed in the previous sections and evaluate their performance compared to competition. All data related to experiments is available online at https://github.com/Alex-Andrv/evo_network_public.

### 4.1 Benchmarks

In the experiments we considered two classes of networks. The first one is formed by random networks of small size generated using the well-known models: the Watts-Strogatz model [52] (WS-networks) and Barabasi-Albert model [53] (BA-networks). We used these networks to construct exact solutions for TSS and evaluate the quality of solutions found by metaheuristic algorithms by comparing them against the exact one. The second class of benchmarks was formed by the fragments of real-world networks taken from the SNAP [32] repository. We used them to compare the considered evolutionary and hybrid algorithms against each other when solving IM and TSS.

#### 4.1.1 Network graphs.

In more detail, for the experiment with small random networks to generate the latter we employed the well-known NetworkX package [54]. For the Watts-Strogatz model we generated networks with 40 vertices, where each node is joined with *k* = 8 neighbors in a ring and each edge has the probability of β=0.5 to be rewired. In the case of Barabasi-Albert model we generated graphs with 50 vertices, and used the value of parameter *m* (the number of edges to attach from a new node) equal to 4, with no initial graph specified (in which case NetworkX starts from a star graph on m+1 nodes).

As for the benchmarks from the SNAP repository, we used a number of networks with several thousand vertices on average. Specifically, we picked facebook_combined (which is a whole network from the ego-Facebook SNAP entry), p2p-Gnutella06_combined, p2p-Gnutella08_combined, wiki-vote and ca-GrQc networks.

The majority of network graphs from SNAP as well as the generated small graphs are undirected, thus we replaced each edge by a pair of arks going in opposite directions.

#### 4.1.2 Arc weights and thresholds.

To generate arc weights $b_{\left(u,v\right)}$ we chose the numbers $B_{\left(u,v\right)}$ (see Sect 1.1) uniformly from a set {1,…,K}, where *K* was chosen depending on the network size. For the small networks the values of $B_{\left(u,v\right)}$ were picked from the range of {1,2} for the Watts-Strogatz networks and from {1,…,5} for the Barabasi-Albert networks. In the case of SNAP networks to reflect the possibility that the influence of some agents may be significantly larger than that of other we picked $B_{\left(u,v\right)}$ from {1,…,1000}.

The vertex thresholds were generated with respect to one of the two different thresholds distributions. In the first case we used equal thresholds for all vertices θ=0.8, while in the second case, the value of *θ* for each vertex was chosen randomly from $\left[\frac{3}{4},1\right]$.

Typically, we encapsulate parameter values in a benchmark name. For example, in benchmark BA_50_4_uni_1-5_const_0.8, BA refers to the Barabasi-Albert model, 50_4 to the network size and the parameter for the graph generator (in this case, the number of edges to attach from a new node to existing nodes). The following block refers to the way the arc weights are chosen: uni_1-5 means that the arc weights were picked in accordance with the uniform distribution from the range {1,…,5} The final block const_0.8 refers to the scheme used to choose thresholds and in the example it employs constant thresholds equal to 0.8 for each vertex.

### 4.2 Comparing to exact solution

The objective of the first experiment was to demonstrate that evolutionary algorithms and the combination of the greedy and evolutionary algorithms given enough resources can produce solutions that are close in size to the exact solution. To show it we used small random networks described in Sect 4.1, so that we were able to find the exact solutions using combinatorial algorithms, in the role of which we employed modern SAT solvers.

Note that in this context it is natural to consider TSS in the following formulation: to find the TS of smallest size that activates at least ≥R network vertices in *M* time steps. To reduce this problem to SAT we used the methods similar to those described in [42] and the PySAT toolkit [55]. As the value of *M* we used the number of vertices in networks, thus in this part of the experiments *M* = *n*. If in the process of solving the algorithm finds a set of size $k^{\prime }$ then the formula is constructed that encodes the existence of a TS of size $k^{\prime }-1$ which activates at least *R* vertices in *M* time steps. If this formula is unsatisfiable for TS of size $k^{\prime }-1$ then the TS of size $k^{\prime }$ is considered to be the exact solution.

We applied to the same networks the combination of the greedy and the evolutionary algorithm, in particular, (1 + 1)-EA. The evolutionary algorithm started either from a set corresponding to all network vertices or from an initial point corresponding to the solution found by the greedy algorithm.

The results of the experiment are presented in Table 1. It is clear from the table that in most cases the hybrid strategy manages to find the solution which is close to optimal, and often does find the optimal solution.

**Table 1:** Results on the comparison of evolutionary and greedy algorithms with exact solution.

Benchmark	SAT (exact)	GrA	(1 + 1)-EA	GrA&(1 + 1)-EA
BA_50_4_uni_1-5_uni_0.75-1	15	18	*16.35*	*15.6*
BA_50_4_uni_1-5_const_0.8	16	19	*17.5*	*17.1*
WS_40_8_0.5_uni_1-2_uni_0.75-1	15	20	*17.65*	*17.1*
WS_40_8_0.5_uni_1-2_const_0.8	16	21	*16.9*	*16.25*

In all cases the value represents the TS size. Evolutionary algorithms were allowed to perform up to 10000 iterations. The results of nondeterministic algorithms are averaged over 20 launches and are presented in cursive.

### 4.3 Solving IM with (1 + 1)-WEA

The goal of the next series of experiments was to see whether the proposed mutation operator in (1 + 1)-WEA really manages to improve the quality of solution in the context of IM, e.g. when we fix the size of the target set and aim to maximize the number of activated networks.

For this purpose, we used the GrA algorithm to find TSS solutions for M=h×n, where h∈{0.5,0.6,0.7,0.8} and *n* is the number of vertices in a network. Recall that similar to above we denote by *k* the size of the constructed TS. Next, we applied (1 + 1)-WEA (with the heuristics to decrease TS size disabled) to see how it performs in solving IM for specific values of *k*. For this purpose, we used the benchmarks based on the SNAP networks described in Sect 4.1.

**Fig 2:**
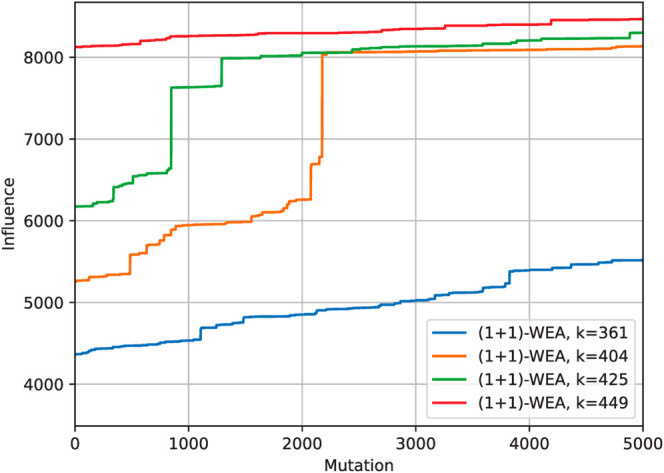
Convergence plots for solving IM for p2p-Gnutella06_uni_1-1000_uni_0.75-1 via (1 + 1)-WEA. The value of *k* corresponds to the size of the target set, the influence (vertical axis) refers to the number of activated vertices in a fixed point, while mutations (horizontal axis) means the number of mutations (iterations) made by the evolutionary algorithm.

In the experiments, we observed that in all cases (1 + 1)-WEA was able to improve the quality of the solution found by the greedy algorithm. The Fig 2 presents the convergence plot for one of the considered benchmarks in which the improvements were better than average. It is clear from the figure that (1 + 1)-WEA is sometimes capable of explosive improvements (see orange line on the plot) that result in significant increases in the quality of the found TS. In many observed cases, however, the behavior is more akin to that presented on red and blue lines: clear improvements, but without sudden large increases in influence.

**Fig 3:**
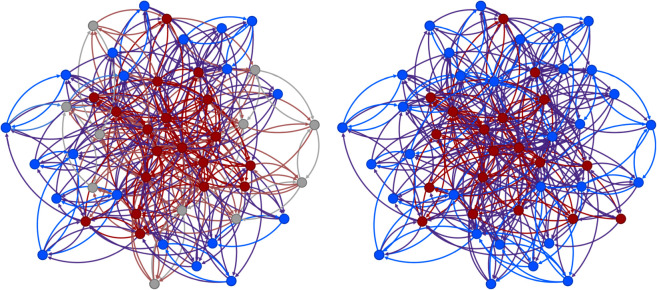
Comparison of the set found when solving IM for *k* = 19 by the greedy algorithm (left) and the one found by (1 + 1)-WEA for a network with *n* = 50 vertices. Vertices from the target set are marked red, active vertices—by blue and inactive vertices are gray. It is clear that the set found using (1 + 1)-WEA manages to activate 11 more vertices out of 50 compared to the set found by the greedy algorithm.

To illustrate the improvements that can be obtained using (1 + 1)-WEA, we generated a small network with 50 vertices, and used greedy algorithm to find a target set of size *k* = 19. The set found by the greedy algorithm is presented in the left part of Fig 3, while that found by the hybrid approach – in the right part of the figure. It is clear that the quality of the set constructed by the proposed approach is significantly higher.

### 4.4 Evaluation of (1 + 1)-WEA for solving TSS

In the final series of experiments we considered SNAP networks and solved TSS using the following algorithms to evaluate their performance: Greedy Algorithm (GrA), (1 + 1)-EA, (1 + 1)-FEA with β=3, Genetic algorithm (GA), (1 + 1)-WEA augmented with heuristics for moving from $\{0,1{\}}_{k}^{n}$ to $\{0,1{\}}_{k-1}^{n}$, which were described in Sect 3: (1 + 1)-WEA_v1, (1 + 1)-WEA_v2, (1 + 1)-WEA_v3. All evolutionary algorithms started from the solution found by the greedy algorithm, which stopped once it found a TS that activates at least R≥0.75×n vertices. Evolutionary algorithms were allowed to perform 10000 iterations: in the case of evolutionary algorithms it was 10000 mutations and in the case of Genetic Algorithm it was 10000 populations of size 10.

From Table 2 one can see that the Genetic Algorithm while having the most resources yields the worst results. We believe that the reason for this behavior is that the crossover and mutation operators in GA need to be specifically tuned to work well with IM/TSS in mind. The analysis shows that the standard crossover operator often produces TSs the Hamming weight of which is too different from that of current best known TS. Therefore, such individuals are typically useless, but they nevertheless consume a lot of allocated resources. From our point of view, adapting crossover for IM and TSS represents a promising direction of future research.

**Table 2:** Results on solving TSS using different evolutionary and greedy algorithms.

	GrA	EA	FEA	GA	WEA_v1	WEA_v2	WEA_v3
Wiki-Vote							
uni_1-1000_const_0.8	3450	3224	3227	3381	3193	**3180**	**3180**
uni_1-1000_uni_0.75-1	3380	3170	3174	3305	3142	3136	**3135**
p2p-Gnutella06							
uni_1-1000_const_0.8	582	490	489	536	400	**393**	397
uni_1-1000_uni_0.75-1	437	376	376	406	319	309	**300**
p2p-Gnutella08							
uni_1-1000_const_0.8	332	268	271	301	233	**216**	219
uni_1-1000_uni_0.75-1	281	227	228	252	185	**176**	**176**
facebook_combined							
uni_1-1000_const_0.8	1526	1428	1426	1486	**1371**	1411	1411
uni_1-1000_uni_0.75-1	1371	1254	1255	1318	**1211**	1243	1243
ca-GrQc							
uni_1-1000_const_0.8	1592	1536	1535	1574	**1496**	1500	1499
uni_1-1000_uni_0.75-1	1475	1413	1412	1451	**1366**	1376	1374

In all cases the value represents the TS size. Evolutionary algorithms were allowed to perform up to 10000 iterations and started from the solution found by the greedy algorithm in all cases, thus we omit this information in column names. We also omit the (1 + 1) part for brevity. The results of nondeterministic algorithms are averaged over 20 launches and rounded. The best result for each benchmark is outlined in bold. The values are rounded to the closest integer.

We computed a Wilcoxon Signed Rank Test to study the (1 + 1)-WEA performance improvement over other methods. It revealed a statistically significant difference in TS size found by different methods, in particular, the results of the proposed WEA algorithm (in any of three variations) significantly differ from others (*p*<0.05), with a large effect size (*r* = 0.886) [56].

**Fig 4:**
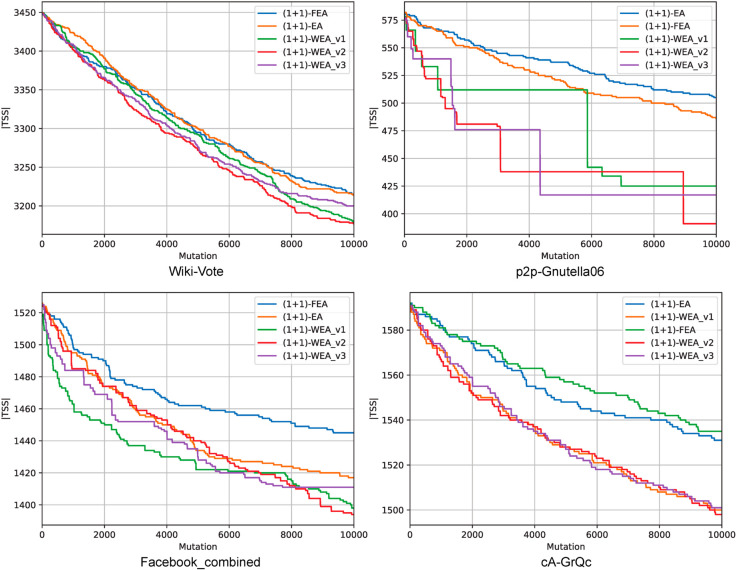
Convergence plots for solving TSS for SNAP networks.

Fig 4 presents convergence plots for a single run of the algorithms for four out of five networks. From both Fig 4 and Table 2 it is clear, that (1 + 1)-EA and (1 + 1)-FEA show comparable results, which are substantially better compared to that of GA. In almost all cases they make it possible to improve the solution found by the Greedy algorithm.

Finally, three versions of (1 + 1)-WEA show the best results overall. An interesting fact is that in the considered test series typically different versions of (1 + 1)-WEA perform better than the others, and this behavior perseveres for different arcs’ weights and vertices’ thresholds. It is natural to assume that they tend to align well with graph structure. Thus, another interesting venue for future research consists in learning to pick the best heuristic based on the effectively computable network properties, such as centrality, etc.

### 4.5 Sensitivity analysis

In this subsection we present the results on the sensitivity analysis of the behavior of the (1 + 1)-WEA_v3 algorithm described in Sect 3 under different values of parameter *Q*. In this series of experiments we used the same networks as in the previous subsection.

Remind, that the v3 heuristic described in Sect 3 attempts to remove from the current target set of size *k* a single vertex, and then launches (1 + 1)-WEA over {0,1}^*k*−1^ to maximize the influence. If it manages to find ${\tilde{\alpha }}^{*}\in \{0,1{\}}^{k-1}:\sigma ({\tilde{\alpha }}^{*})\geq R$ then this ${\tilde{\alpha }}^{*}$ becomes the current best known target set.

When attempting to transition from TS of size *k* to TS of size *k*–1 using (19), we want to compute the value $wt_{H}(\overset{―}{\alpha })-wt_{H}(\overset{―}{\alpha_{v}})$ for as few TS candidates as possible, thus we pic the first *Q* of them when sorted by the value of (18).

We tested the values Q∈{25,50,75,100,125}, the corresponding results are presented in Table 3. It is clear from them that the value of *Q* has quite little impact on the quality of the obtained solutions in most cases.

**Table 3:** Results on solving TSS using (1 + 1)-WEA_v3 with different values of *Q.*

	Q=25	Q=50	Q=75	Q=100	Q=125
Wiki-Vote					
uni_1-1000_const_0.8	**3175**	3180	3181	3181	3179
uni_1-1000_uni_0.75-1	3138	3135	**3131**	3138	3139
p2p-Gnutella06					
uni_1-1000_const_0.8	399	**397**	396	396	400
uni_1-1000_uni_0.75-1	309	**300**	315	303	310
p2p-Gnutella08					
uni_1-1000_const_0.8	223	219	218	**217**	221
uni_1-1000_uni_0.75-1	179	176	**175**	177	178
facebook_combined					
uni_1-1000_const_0.8	**1407**	1411	1411	**1407**	1415
uni_1-1000_uni_0.75-1	**1235**	1243	**1235**	1246	1242
ca-GrQc					
uni_1-1000_const_0.8	**1497**	1499	1500	1498	**1497**
uni_1-1000_uni_0.75-1	**1375**	**1374**	1376	1377	1375

In all cases the value represents the TS size. The results are averaged over 20 launches and rounded. The best result for each benchmark and value of *Q* is outlined in bold.

We used Wilcoxon Signed Rank test to see whether any of the values of *Q* shows statistically significant performance improvement over the others, but on the considered benchmarks the amount of data was not sufficient enough to reject a null hypothesis about the equality of medians between every pair of values.

**Fig 5:**
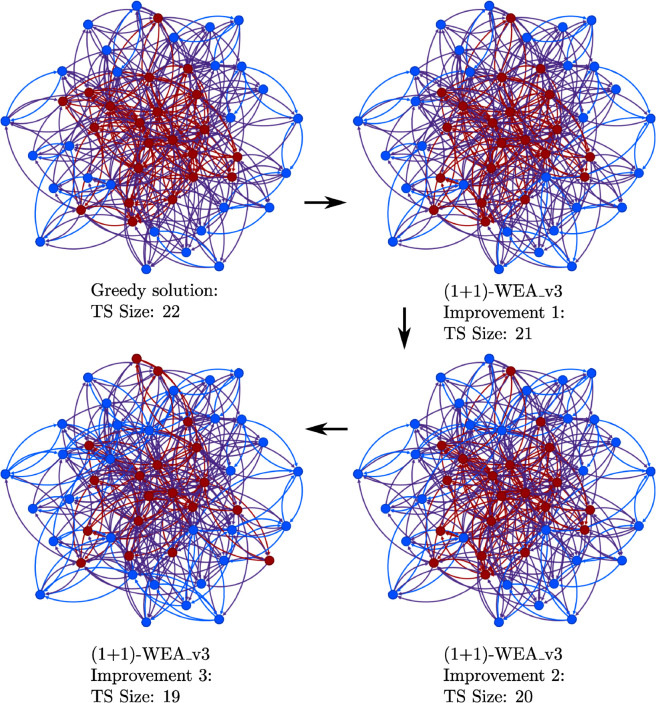
Illustration of how (1 + 1)-WEA_v3 improves the target set when solving TSS. The goal is to activate all vertices in the network. The network with 50 vertices is the same as in Fig 3. The first target set (left) is found by the greedy algorithm. The next three target sets are found consecutively by our algorithm. Vertices from the target set are marked red, active vertices – by blue.

On Fig 5 we show how the (1 + 1)-WEA_v3 algorithm improves the target set during several iterations. Here, we used the same network as in Fig 3.

### 4.6 Analysis of practical efficiency

Let us say a few words about the practical complexity of the employed algorithms. It is clear that a single mutation is performed by (1 + 1)-EA and (1 + 1)-WEA in time *O*(*n*), where *n* is the size of the mutated vector. Theoretical upper bounds on the number of mutations until the considered algorithms reach a global extrema can be found in Sect 2. In practice, the typical approach is to limit the number of mutations by some constant. In our experiments, we used the limits from 1000 to 10000 mutations. Usually, within the allocated mutation budget, the algorithms manage to substantially improve the initial value of the target function found by the greedy algorithm.

As for the memory, the described algorithms do not have any specific requirements: it is clear that the total amount of memory used by the (1 + 1)-EA and (1 + 1)-WEA algorithms from start to the moment they find a global extrema does not exceed *O*(*n*). Indeed, they basically store in the memory the best point, corresponding to the Best Known Value of a considered function in that point, and the current point. Also note, that the functions we optimized in the present paper are relatively cheap, compared to e.g. functions from [57,58]. Therefore, for these functions we can see that the (1 + 1)-evolutionary strategy works significantly better compared to more complex genetic algorithms operating with populations of size greater than one. Thus, for the considered functions the more effective strategy is the one that processes more points per the unit of time.

## 5 Related work

As it was mentioned above, to the best of our knowledge, the paper [33] by Stewart Kauffman was the first work in which networks were used to define dynamical systems with discrete time and finite number of different states. Also, in that work it was proposed to associate Boolean functions with network vertices, defining functions via the truth tables. The information diffusion model considered in [33] is exactly the same as the DLTM in the variant described in our paper. It was used there to describe possible interactions in biological systems known as Gene Regulatory Networks.

Later, the term “Kauffman Networks” was used in a number of works, e.g. [59,60], that studied different critical phenomena in these networks, such as distributions of the length of cycles produced by these networks, statistical characteristics for some kinds of clusters (called modules in [60]), as well as the analysis of similarities with the percolation theory (in this point the authors of [59,60] refer to [61]). Papers [62,63] studied the cyclic structure of state transition graphs for DDS induced by Kauffman Networks. In particular, in [62] to find cycles of small length they use Binary Decision Diagramns (BDD) – the well-known structute actively used in symbolic verification [64].

The idea to define the weight function of network vertices using thresholds was apparently first proposed by Mark Granovetter in [26]. In that paper, a number of examples that demonstrate the correspondence between the threshold model of activation dynamics and the so-called conforming behavior which is often observed in real life collectives, was provided. We would like to mention several further works which developed the ideas from [26], in particular [65–67], etc.

The paper [4] for more than 20 years remains the central work in the context of maximizing influence in networks and continues to stimulate novel results in this area. It introduced all the main notions related to influence maximization. At the same time, the authors of [4] note that they were largely inspired by the problems considered in [68]. The main models under which IM is usually considered, in particular, Linear Threshold Model and Independent Cascade Model, were also proposed in [4]. The paper also showed that for the nondeterministic variants of these models there exists a polynomial algorithm that approximates the solution of IM with approximation factor 1–*e*^−1^. However, for the deterministic variants of both models it gave strong arguments on their inapproximability.

IM is one of the most actively researched problems in network science in the last 20 years: see [4–21]-[25,68],etc. Let us briefly comment only on the results of the most cited of the mentioned works. Thus, [6] is a direct development of [4]: in particular, they studied in detail the so-called decreasing cascade model, the basic properties of which were formulated in [4] in form of hypotheses. The authors of the paper [69] state that the approach they proposed based on the meticulous analysis of the submodularity property outperforms the basic greedy algorithm from [4] in hundreds of times on some networks. In [7] there have been studied the questions related to influence diffusion in social networks by aggregating the groups of users by the topics of their interests, the corresponding approach is called Topical Affinity Propagation. In [8] they proposed the strategy of Two-phase Influence Maximization which makes it possible for the non-deterministic Independent Cascade model to significantly speed up the standard greedy algorithm without falsifying the approximability guarantees of the obtained solutions. The paper [9] studies the problem of activating the network by means of top-K influential vertices. As the authors mention in text, their algorithm is aimed at solving the problem of activating mobile social networks with a large number of participants. Several works, such as [11,12], proposed improvements to the greedy algorithm and some additional centrality-based heuristics, which make it possible to increase the dimension of effectively activated networks to millions of vertices. In [15] the authors studied nontrivial questions related to the structural complexity of IM, in particular, they showed the #P-hardness of exact influence computation in the context of the Linear Threshold model. Some peculiarities of the structure of real-world information networks that pass through themselves colossal volumes of information (millions of blogs and news articles) were studied in [17]. A time-constrained variant of IM was apparently first considered in [19]. The Influence Maximization for a network which changes with time was analyzed in [20] In [21] it was showed that taking into account the information about location may play an important role when maximizing the influence in real-world marketing strategies. The robustness of IM solutions under uncertainty of some network parameters (specifically, probabilities assigned to edges) was studied in [24].

Surprisingly, the papers dealing with IM under DLTM are significantly fewer in number compared to that for LTM, despite the fact that the ideas how one can solve IM under DLTM using heuristic and metaheuristic algorithms look natural. The greedy algorithm for DLTM which we use to construct an initial solution of IM and TSS was described in [37]. Several subsequent works used some generalizations of this algorithm: [70,71], etc. The paper [70] considered the targeted and budgeted variant of IM, in which the profits and costs of activating vertices were added, and proposed the modification of the greedy algorithm. In [71] there was considered a time-constrained variant of IM under DLTM. The intention to demonstrate that it is possible to solve IM and TSS under DLTM by combining greedy heuristics and evolutionary optimization became our main motivation for writing the present paper.

The TSS problem was first implicitly formulated in [4]. The formulation that we use in our paper was employed in [72] and [35]. The structural complexity of IM and TSS was first evaluated in [4]. As we already mentioned, there are approximation algorithms with constant factor for IM under nondeterministic LTM (when influence is defined as an expectation (3)), meanwhile, under DLTM the solution of IM cannot be efficiently approximated if P≠NP. It was noted in [35], that the intractability of approximation of TSS under DLTM follows from this fact. A number of further results related to the complexity of TSS was also presented in [35]. In particular, it was shown that some non-monotonic variants of TSS are #P-hard.

As we mentioned above, both IM and TSS under DLTM can be solved exactly using modern combinatorial algorithms. However, the dimension of the problems with which such algorithms are able to successfully deal with is quite small: typically in the dozens or at most hundreds of vertices. In [42,73] the algorithms for solving the Boolean satisfiability problem (SAT) were used to solve TSS. Note, that in [42,74–76] the algorithms based on SAT or on Binary Decision Diagrams (BDD) were also used to find attractors (cycles) of small length in State Transition Graphs of Kauffman networks and networks with more elaborate weight functions modeling dynamic processes in gene networks. It is also worth mentioning, that the standard network activation model considered in the majority of related works (including the present paper) implies the monotonic scenario, in which DDS has only attractors of length 1 (i.e. fixed points). This scenario corresponds to the so-called conforming behavior in terms of [26]. In paper [42] it was showed that replacing functions $f_{v}(t\;+\;1)$ of the kind (1) by functions of the kind ${\tilde{f}}_{v}(t\;+\;1)=1-f_{v}(t\;+\;1)$, corresponding to anti-conforming behavior, results in DDS with STG the structure of which is significantly different from the structure of STG for the conforming scenario.

We would like to specifically note the series of works in which the Discrete Dynamic Systems of the same kind as the ones considered in our paper were studied from the point of view of cellular automata. In particular, in [77] they showed that the general reachability problem for models close to DLTM is PSPACE complete. The works [78,79] studied a number of problems related to reachability and specifically showed the NP-completeness of the general Predecessor Existence problem in the context of DDS similar to Synchronous Boolean Networks.

The networks we use in the paper can be broadly divided into two main classes. First, these are network constructed using the random graph models, such as the Watts-Strogatz model [52] and Barabasi-Albert model [53]. Second, there are fragments of real-world networks available in the SNAP repository [32].

The evolutionary and genetic algorithms are among the most popular tools for metaheuristic optimization [45]. The (1 + 1)-EA algorithm can be viewed as the simplest implementation of the idea of evolutionary computations. Nevertheless, this algorithm has a lot of nontrivial mathematical properties studied in e.g. [49,51,80–83], etc. In particular, as we mentioned above, in [49] there was proposed a natural approach to the study of the behavior of (1 + 1)-EA on a wide class of pseudo-Boolean functions. For a number of artificially constructed functions in [49] it was shown that (1 + 1)-EA can be both either very effective (e.g. for ONEMAX function) or vice versa. For an arbitrary pseudo-Boolean function without any analytical specification (1 + 1)-EA is highly ineffective in the context of the bounds from [49]. Note, that similar results were presented in paper [84]. In [51] an approach was proposed in the context of which (1 + 1)-EA changes its mutation rate according to some probabilistic distribution. A similar idea was used earlier in [80]. The (1 + 1)-EA variant from [51] has a significantly better upper bound on its complexity compared to the original (1 + 1)-EA. In papers [83] and [85] variants of (1 + 1)-EA were described with the complexity upper bound significantly smaller than that of the classic algorithm.

The variant of (1 + 1)-EA which is based on the $\mu_{k}^{n}$ operator was first described in the conference paper [86]. In that paper this algorithm was used to find the so-called *ρ*-backdoors described in [87] which in essence represent a probabilistic generalization of backdoors for SAT introduced in [88]. In the present paper we performed the extended analysis of the properties of the algorithm from [86] and significantly improved the upper bound of its complexity for pseudo-Boolean functions without any analytical specification. One of the contributions of the present paper lies in adapting (1 + 1)-WEA for solving IM and TSS.

To the best of our knowledge, the conference paper [89] has the first examples of the application of evolutionary algorithms to solving IM under DLTM. Our two conference papers [90,91] present the preliminary results on solving TSS using different evolutionary algorithms, including that from [51,86] and [85].

## 6 Discussion, conclusion and future work

In the present paper we considered two well-known combinatorial problems for Boolean networks: Influence Maximization and Target Set selection. We studied these problems in the context of the Deterministic Linear Threshold Model, while the majority of papers on the topic consider nondeterministic models, first formally presented in [4]. This choice is motivated by a number of factors. First, DLTM agrees well with many practical examples, the nature of which was described by Mark Granovetter in [26]. Second, our goal was to employ for solving IM and TSS the metaheuristic algorithms used in pseudo-Boolean optimization and specifically evolutionary algorithms. It should be noted that the described computational schemes may be applied to non-deteministic LTM from [4], but in this case it makes sense to implement them in parallel environments, since computing the fitness function under LTM is much more expensive compared to DLTM. Indeed, for large networks the value (3) can only be estimated using the Monte Carlo method [92], and the accuracy of this estimation improves with the increase of the size of the random sample. From our point of view, the study of how the network size influences the accuracy of computing influence in the sense of (3) is an important problem which we plan to consider in the nearest future. In particular, we believe that the results presented in this paper look promising in that the corresponding algorithms for solving IM and TSS under LTM can be built upon the parallel versions of the algorithms proposed in this paper.

The use of neural networks for solving IM and TSS looks promising. This direction fits into a general area where neural networks are used to tackle NP-hard problems. It grew in popularity in recent years thanks to the ability of neural networks to perform the role of universal approximators. The examples of such an approach can be found in papers [93–97], etc. Some of the works, in particular [98–100], and some others, show the possible applications of neural networks for solving IM and TSS. However, the cited papers typically either consider an Independent Cascade Model or the Linear Threshold Model in their standard non-deterministic variants from [4]. Our attempts to apply the tools presented in these articles to DLTM did not yield good results, since we observed the effects mentioned in [94], e.g., various “bottlenecks”, in particular, the lack of robustness of the neural network models when fed with data only implicitly related to the problems they were designed to solve. As a result, to construct the target sets similar in their effectiveness to that found by our hybrid approach, one would need to employ a very resource-intensive training stage. Nevertheless, we believe that an approach to solving IM via Neural Networks is very promising and we plan to address it in our future works. In particular, we believe that Binarized Neural Networks [101] suit well to solving IM and TSS in the context of DLTM and plan to conduct corresponding research in the near future. In particular, we plan to study the possibilities in combining the proposed evolutionary algorithms with binarized neural networks, specifically to use the latter to improve the initial solutions to IM and TSS found by greedy heuristics.
